# Oxidative Stress Facilitates IFN-γ-Induced Mimic Extracellular Trap Cell Death in A549 Lung Epithelial Cancer Cells

**DOI:** 10.1371/journal.pone.0162157

**Published:** 2016-08-30

**Authors:** Chiou-Feng Lin, Chia-Ling Chen, Shun-Yi Chien, Po-Chun Tseng, Yu-Chih Wang, Tsung-Ting Tsai

**Affiliations:** 1 Department of Microbiology and Immunology, School of Medicine, College of Medicine, Taipei Medical University, Taipei 110, Taiwan; 2 Graduate Institute of Medical Sciences, College of Medicine, Taipei Medical University, Taipei 110, Taiwan; 3 Translational Research Center, Taipei Medical University, Taipei 110, Taiwan; University of Alabama at Birmingham, UNITED STATES

## Abstract

We previously demonstrated that IFN-γ induces an autophagy-regulated mimic extracellular trap cell death (ETosis) in A549 human lung cancer cells. Regarding reactive oxygen species (ROS) are involved in ETosis, this study investigated the role of oxidative stress. After IFN-γ stimulation, a necrosis-like cell death mimic ETosis occurred accompanied by the inhibition of cell growth, aberrant nuclear staining, and nucleosome release. ROS were generated in a time-dependent manner with an increase in NADPH oxidase component protein expression. STAT1-mediated IFN regulatory factor-1 activation was essential for upregulating ROS production. By genetically silencing p47^phox^, IFN-γ-induced ROS and mimic ETosis were significantly attenuated. This mechanistic study indicated that ROS may mediate DNA damage followed by histone H3 citrullination. Furthermore, ROS promoted IFN-γ-induced mimic ETosis in cooperation with autophagy. These findings further demonstrate that ROS regulates IFN-γ-induced mimic ETosis in lung epithelial malignancy.

## Introduction

The cytokine IFN-γ is crucial for innate and adaptive immunities, which promote immunomodulation and anti-microbe and anticancer activities [1]. An IFN-γ deficiency in mice presents as inadequate tumor immunosurveillance, which results in the spontaneous development of lymphoma, sarcoma, and lung epithelial malignancies and accelerated carcinogen-induced tumorigenesis [2, 3]. To induce growth inhibition and cell cycle arrest, IFN-γ stimulation transcriptionally induces p21, p27, and p53 expression [4–7]. To induce cell death, IFN-γ increases Fas and Fas ligand expression, which facilitate cellular sensitivity to apoptotic stimuli [8]. Additionally, IFN-γ induces autophagy-related growth inhibition and cell death [9, 10]. Our current study demonstrates autophagy-regulated necrotic effects from IFN-γ in lung adenocarcinoma cells [11]. We propose that both direct and indirect effects of IFN-γ are involved in the induction of IFN-γ-mediated cytotoxicity.

IFN-γ triggers signal transduction via the IFN-γ receptor (IFNGR). Through IFNGR dimerization, IFN-γ activates Janus-associated kinase (JAK) 1/2 to stimulate signal transducers and activators of transcription (STAT) 1 phosphorylation followed by nuclear translocation. Upon STAT1 activation, various IFN-regulated factors (IRFs) are transactivated to regulate IFN-γ bioactivities [1]. In addition to JAK1/2-STAT1-IRF-1 signaling, the immunity-related GTPase family M protein (IRGM) mediates IFN-γ-induced autophagy and autophagy-mediated mimic extracellular trap cell death (ETosis) in A549 human lung adenocarcinoma cells [11]. ETosis—a novel form of cell death—was previously identified in immune cells in response to phorbol myristate acetate, cytokines, chemokines, bacteria, protozoa, and viruses [12]. During the ETosis process, chromatin externalization induces immune defenses and inflammation [13].

Various molecules are either independently or cooperatively involved in ETosis regulation. Chromatin decondensation is generally regulated by peptidyl arginine deiminase 4 (PAD4)-mediated histone hypercitrullination [14]. Ca^2+^-dependent PAD4 converts histone arginine side chains to citrulline through deimination [15]. Additionally, NADPH oxidase-regulated reactive oxygen species (ROS) are essential for PAD4 activation and ETosis [16, 17]. Furthermore, autophagy also contributes to ETosis through an unknown mechanism in neutrophils [17]. Herein, we demonstrate that IFN-γ induces autophagy-based Fas-associated protein with death domain/caspase-8 activation, resulting in caspase-regulated DNA damage followed by PAD4 activation and ETosis [11]. In this study, the involvement of ROS generation in IFN-γ-induced mimic ETosis in lung epithelial malignancy was investigated.

## Materials and Methods

### Cells, culture condition, and reagents

A549 (CCL185, ATCC) human lung epithelial adenocarcinoma cells were grown in DMEM (Invitrogen Life Technologies, Rockville, MD) supplemented with 10% heat-inactivated FBS (Invitrogen Life Technologies), 50 U/ml penicillin and 50 μg/ml streptomycin. Human recombinant IFN-γ was obtained from PeproTech (Rocky Hill, NJ). Mouse monoclonal antibody specific for β-actin was obtained from Sigma-Aldrich (St. Louis, MO). Alexa Fluor 488-labeled anti-mouse and anti-rabbit IgG were obtained from Invitrogen (Carlsbad, CA). Antibodies against goat conjugated with HRP, p47^phox^, p67 ^phox^, and gp91^phox^ were purchased from Santa Cruz Biotechnology (Santa Cruz, CA). Antibodies against rabbit conjugated with HRP and phospho-γ-H2AX (S139) were obtained from Millipore (Billerica, MA). Antibodies against citrullinated histone H3 (citrulline 2+8+17) and histone H3 were obtained from Abcam (Cambridge, MA). Antibody against LC3 was purchased from MBL International (Woburn, MA).

### Transmission electron microscopy

Analysis of cell morphological change was performed by using transmission electron microscopy (JEOL JEM-1200EX, Tokyo, Japan). The cell preparation and experimental procedures were carried out according to the previous study [11].

### Cell viability and cytotoxicity assays

A microplate reader (SpectraMax 340PC; Molecular Devices Corporation, Sunnyvale, CA, USA) was used to determine cell proliferation using a colorimetric assay (Cell Counting Kit-8; Dojindo Molecular Technologies, Kumamoto, Japan) according to the manufacturer’s instructions. To evaluate cytotoxicity, lactate dehydrogenase (LDH) activity was assayed using a colorimetric assay (Cytotoxicity Detection kit; Roche Diagnostics, Lewes, UK) according to the manufacturer’s instructions. The data were analyzed using Softmax Pro software (Molecular Devices Corporation). The relative proliferation rate was calculated by normalization to the control group. Further cell viability was also assessed using the trypan blue exclusion test and was calculated as follows: relative viability (%) = (viable cell number in IFN-γ treatment group/viable cell number in control group) × 100.

### Image analysis

Cells were fixed in 3.7% formaldehyde in PBS and then incubated with primary antibodies. The samples were washed with PBS twice and then incubated with Alexa Fluor 488-labeled secondary antibodies. The antibodies were against phospho-γ-H2AX (S139). 4',6-diamidino-2-phenylindole (DAPI; Sigma-Aldrich) was used for nuclear staining. Cells were observed under a fluorescent microscope (BX51; Olympus, Tokyo, Japan) or a laser-scanning confocal microscope (Digital Eclipse Clsi-ready; Nikon).

### Enzyme-linked immunosorbent assay (ELISA)

For detection of extracellular nucleosome as shown by the previous study [18, 19], the concentrations of DNA-histone complex (nucleosomes) in cultured supernatant were determined with ELISA. Cell-conditioned culture medium was co-incubated with biotin-labeled anti-histone antibody and peroxidase-conjugated anti-DNA antibody (Cell Death Detection ELISA^PLUS^; Roche Diagnostics) at room temperature. After rinsing three times, peroxidase substrate was added. A microplate reader (SpectraMax 340PC) was used to measure absorbance at 405 nm, and the data were analyzed using Softmax Pro software.

### Intracellular ROS assay

Cells were exposed to 20 μM 5-(and-6)-chloromethyl-2',7'-dichlorodihydrofluorescein diacetate, acetyl ester (CM-H_2_DCFDA) (Invitrogen) for 1 h. Fluorometric determination of intracellular ROS generated by the cells were detected using a fluorescent microplate reader (Molecular Device, SpectraMax GeminiXS) and the data were analyzed using Softmax Pro software.

### Western blot analysis

Total cell extracts were lysed using a lysis buffer containing 1% Triton X-100, 50 mM Tris, pH 7.5, 10 mM EDTA, 0.02% NaN_3_, and a protease inhibitor cocktail (Roche Diagnostics, Mannheim, Germany) and then subjected to 10% SDS-PAGE and transferred to polyvinylidene difluoride membranes (Millipore) using a semidry electroblotting system. After blocking with 5% skim milk in PBS, the membranes were incubated with a 1/1000 dilution of primary antibodies and then incubated with a 1/5000 dilution of HRP-conjugated secondary antibodies followed by soaked in an ECL solution (PerkinElmer Life and Analysis Science, Boston, MA) and then exposed to film (BioMax; Eastman Kodak, Rochester, NY). The relative signal intensities were quantified using ImageJ software (version 1.41o) from W. Rasband (National Institutes of Health, Bethesda, MD).

### Lentiviral-based RNA interference and transfection

The short hairpin RNAs (shRNA) clones, including STAT1 (TRCN0000004265, target sequence: CCCTGAAGTATCTGTATCCAA), IRF-1 (TRCN0000014668, target sequence: CGTGTGGATCTTGCCACATTT), and p47^phox^ (clone 1 TRCN0000256331, target sequence: CCATTGCCAACTACGAGAAGA; clone 2 TRCN0000256332, target sequence: TGTACATGTTCCTGGTGAAAT), gp91^phox^ (TRCN0000046083, target sequence: GCCTATATGATCTGCCTACAT), were obtained from the National RNAi Core Facility located at the Institute of Molecular Biology/Genomic Research Center, Academia Sinica, Taiwan. The lentivirus constructs were prepared from the RNAi Core of Research Center of Clinical Medicine, National Cheng Kung University Hospital. Cells were transduced using the recombinant lentivirus at an appropriate multiplicity of infection in complete growth medium supplemented with 8 μg/ml polybrene (Sigma-Aldrich). After transduction for 24 h, puromycin (Calbiochem, San Diego, CA) was used to select cells for at least one week.

### Luciferase reporter assay

Cells were transiently co-transfected with an IRF-1 promoter-driven luciferase reporter (0.5 μg) and 0.01 μg of Renilla luciferase-expressing plasmid (pRL-TK; Promega, Madison, WI). Transfections were performed using the GeneJammer reagent (Stratagene, La Jolla, CA). Twenty-four hours after transfection followed by the additional treatment of IFN-γ, cells were lysed and then harvested for luciferase and Renilla measurements using a luciferase system (Dual-Glo; Promega). The firefly luciferase activity in each lysate was normalized to the Renilla luciferase activity.

### Plasmid transfection

Cells (3 × 10^4^) were transfected with 2 μg of pGFP-C1-LC3 or control vector using TurboFect transfection reagent (Thermo Scientific, Waltham, MA) to detect the formation of autophagosomes. The formation of punctate GFP-LC3 was detected under a fluorescent microscope (IX71, Olympus, Tokyo, Japan). 4',6-diamidino-2-phenylindole (DAPI; Sigma-Aldrich) was used to perform nuclear staining. The percentage of cells with punctuated GFP-LC3 was calculated.

### Statistical analysis

Statistical analysis was performed using Student’s two-tailed unpaired t-test or one-way ANOVA in GraphPad Prism version 5 (La Jolla, CA). Values are expressed as the means ± standard deviation (SD). A *P*-value was under 0.05, results were considered statistically significant.

## Results

### IFN-γ induces cell growth inhibition and cytotoxicity accompanied by mimic ETosis in the A549 lung epithelial cancer cell line

**Fig 1:**
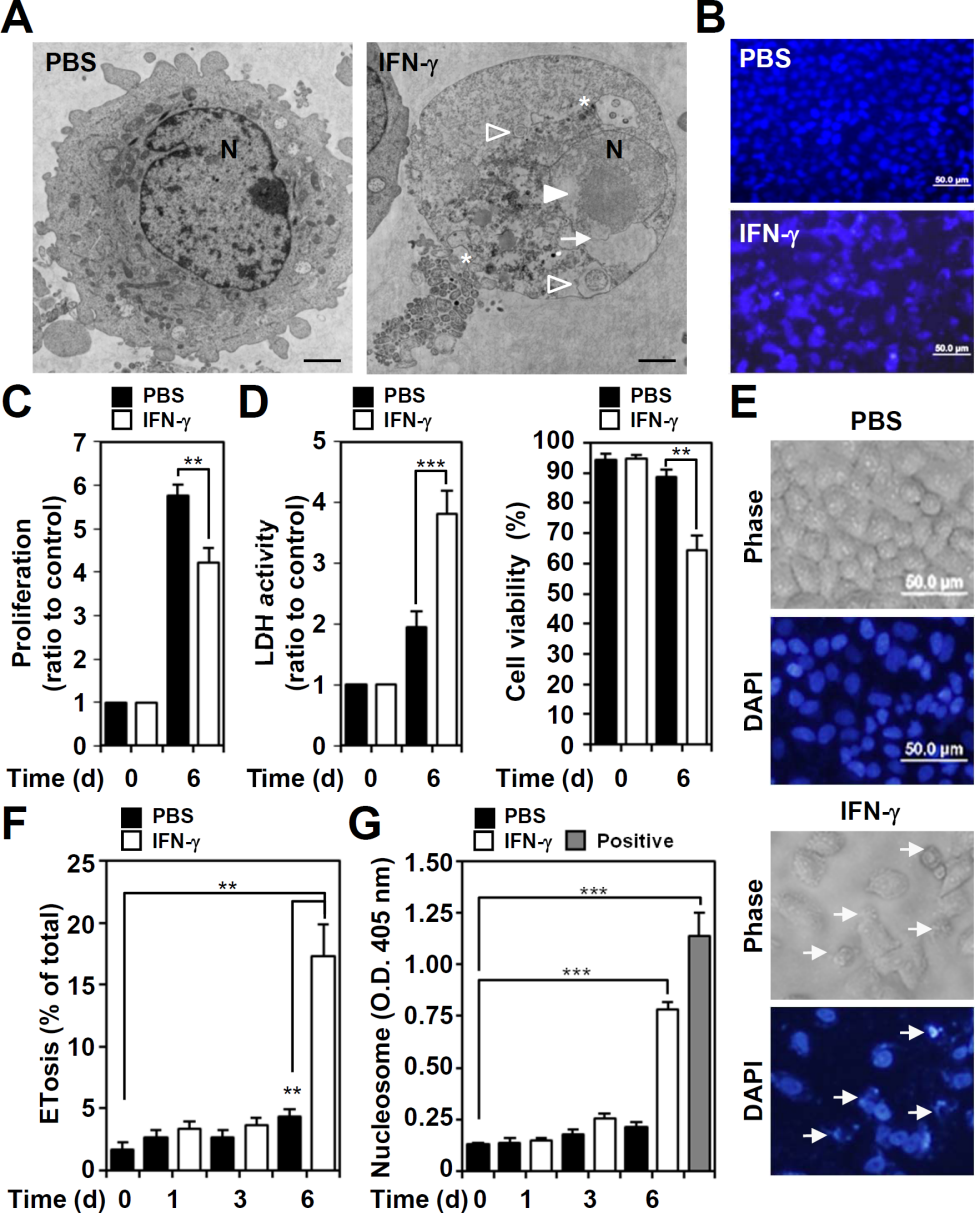
Exogenous IFN-γ causes cell proliferation inhibition and cytotoxicity characterized by a mimic ETosis. (A) Analysis of transmission electron microscopy showed cytoplasmic vacuolization (stars), autolysosome accumulation (empty arrowheads), chromatin decondensation (filled arrowhead), and nuclear membrane disruption (arrow) in IFN-γ (10 ng/ml)-treated A549 cells for 6 days. N, nuclear. Scale bar, 500 nm. (B) Representative DAPI-based nuclear staining followed by fluorescence microscopic analysis showed a mimic ETosis (arrows) in IFN-γ (10 ng/ml)-treated A549 cells 6 days post-treatment. Cell proliferation (C), cell cytotoxicity (D, left), and viability (D, right) were measured in IFN-γ (10 ng/ml)-treated A549 cells for 6 days, and the data are presented as the mean ± SD of three independent experiments, which are shown as the fold change compared to the normalized value of the control or as the percentage of change. **P < 0.01 and ***P < 0.001. (E) Representative morphological change in IFN-γ (10 ng/ml)-treated A549 cells 6 days post-treatment showed a mimic ETosis (arrows) as indicated by DAPI-based nuclear staining followed by fluorescence microscopic analysis. (F) The percentages of cells with mimic ETosis for the indicated time are shown as the mean ± SD of three independent experiments. **P < 0.01. (G) ELISA was used to determine the levels of the extracellular nucleosomes, as a marker of ETosis, in the cell supernatants of IFN-γ (10 ng/ml)-treated cells for the indicated time. The optical density (O.D.) data for the nucleosomes are shown as the mean ± SD of three independent experiments. ***P < 0.001. The empty bar indicates the positive control obtained from the kit. PBS was used as the control for all experiments.

IFN-γ treatment causes a mimic ETosis in A549 cells, characterized with bubble formation followed by an increase in membrane permeability and in chromatin release from the nucleus to the extracellular space [11]. Image analysis of transmission electron microscopy, the mimic ETosis characteristics such as cytoplasmic vacuolization, autolysosome accumulation, chromatin de-condensation, and nuclear membrane disruption were shown in IFN-γ-stimulated A549 cells (Fig 1A). DAPI-based nuclear staining showed that IFN-γ caused extracellular chromatin release from the nuclei (Fig 1B). IFN-γ may promote anticancer activity by triggering cell growth inhibition and cytotoxicity [1]. Cell proliferation assays showed that IFN-γ significantly (*P* < 0.01) inhibited lung cancer A549 cell proliferation 6 days after treatment (Fig 1C). LDH assays and trypan blue exclusion tests showed that IFN-γ caused significant cytotoxicity in A549 cells (*P* < 0.001) (Fig 1D). Using a kinetic model, nuclear staining by DAPI showed IFN-γ-induced mimic ETosis (Fig 1E). At 6 days post-treatment, a significant increase in mimic ETosis was observed (*P* < 0.01, Fig 1F). To confirm a mimic ETosis induced by IFN-γ, our previous works [11] have used more methods, including electronic microscopic observation, lamin A/C degradation, nucleosome release, autophagy-dependent manner, histone 3 hypercitrullination, PAD4 inhibition, to show a mimic ETosis induced by IFN-γ. In this study, we further examined extracellular nucleosome generation using ELISA while the presence of extracellular histone and DNA complex is generally shown in ETosis [11, 18, 19]. The results demonstrated that IFN-γ significantly (*P* < 0.001) induced nucleosome release in a time-dependent manner (Fig 1G). These results show that IFN-γ causes mimic ETosis in A549 cells.

### ROS generation in IFN-γ-stimulated A549 cells

**Fig 2:**
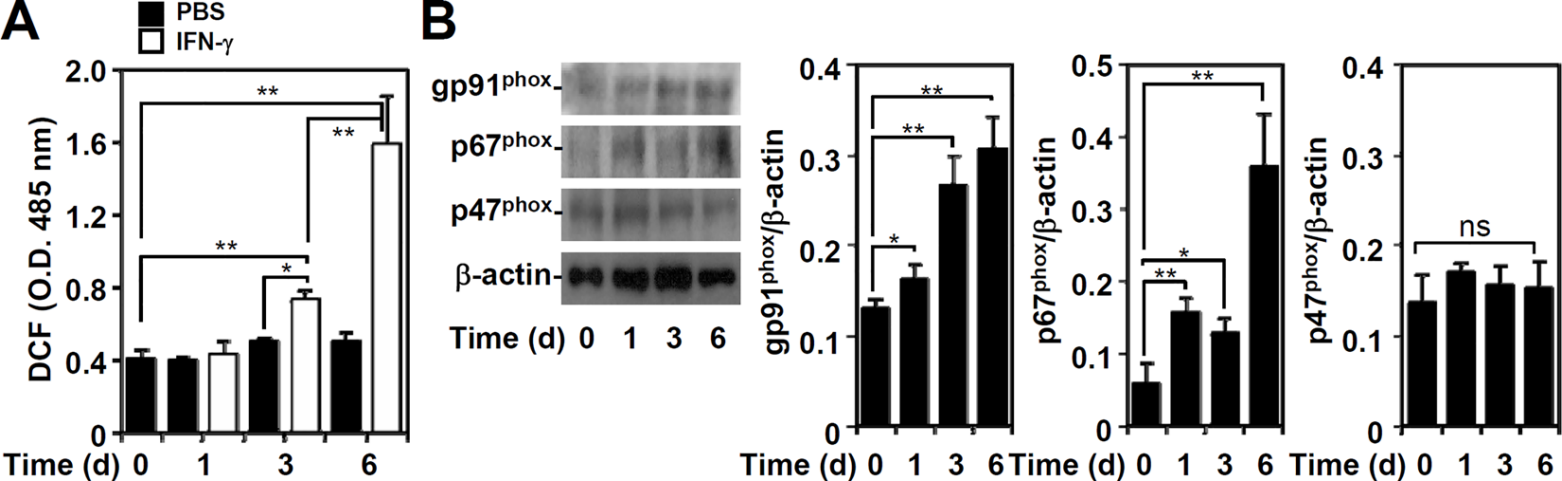
IFN-γ causes ROS generation and NADPH oxidase expression. (A) ROS generation in IFN-γ (10 ng/ml)-treated A549 cells was determined by CM-H_2_DCFDA staining followed by analysis using a fluorescent plate reader for the indicated time. The data are presented as the mean ± SD of triplicate cultures and are shown as the relative optical densities (O.D.). *P < 0.05 and **P < 0.01. PBS was used as a control. (B) Representative Western blotting shows gp91^phox^, p67^phox^, and p47^phox^ expression in IFN-γ (10 ng/ml)-treated A549 cells for the indicated time. β-actin was used as an internal control. The relative ratios of the measured proteins and β-actin are also shown as the mean ± SD of three independent experiments. *P < 0.05 and **P < 0.01. ns, not significant.

To characterize potential mechanisms of IFN-γ-induced cell death through ETosis, we and others have demonstrated that autophagy is critical for inducing ETosis in neutrophils [17] and A549 cells [11]. In addition to autophagy, ROS is also necessary for ETosis in neutrophils [16]. In IFN-γ-treated A549 cells, ROS generation significantly (*P* < 0.01) increased in a time-dependent manner (Fig 2A). Furthermore, IFN-γ also significantly (*P* < 0.01) increased the expression of the NAPDH oxidase subunits gp91^phox^ and p67^phox^, but it did not increase p47^phox^ (Fig 2B), which is consistent with previous studies [20, 21]. These results show that IFN-γ stimulation induces NAPDH oxidase expression and ROS generation.

### Effects of IFN-γ-activated STAT1-IRF-1 on IFN-γ-induced ROS generation

**Fig 3:**
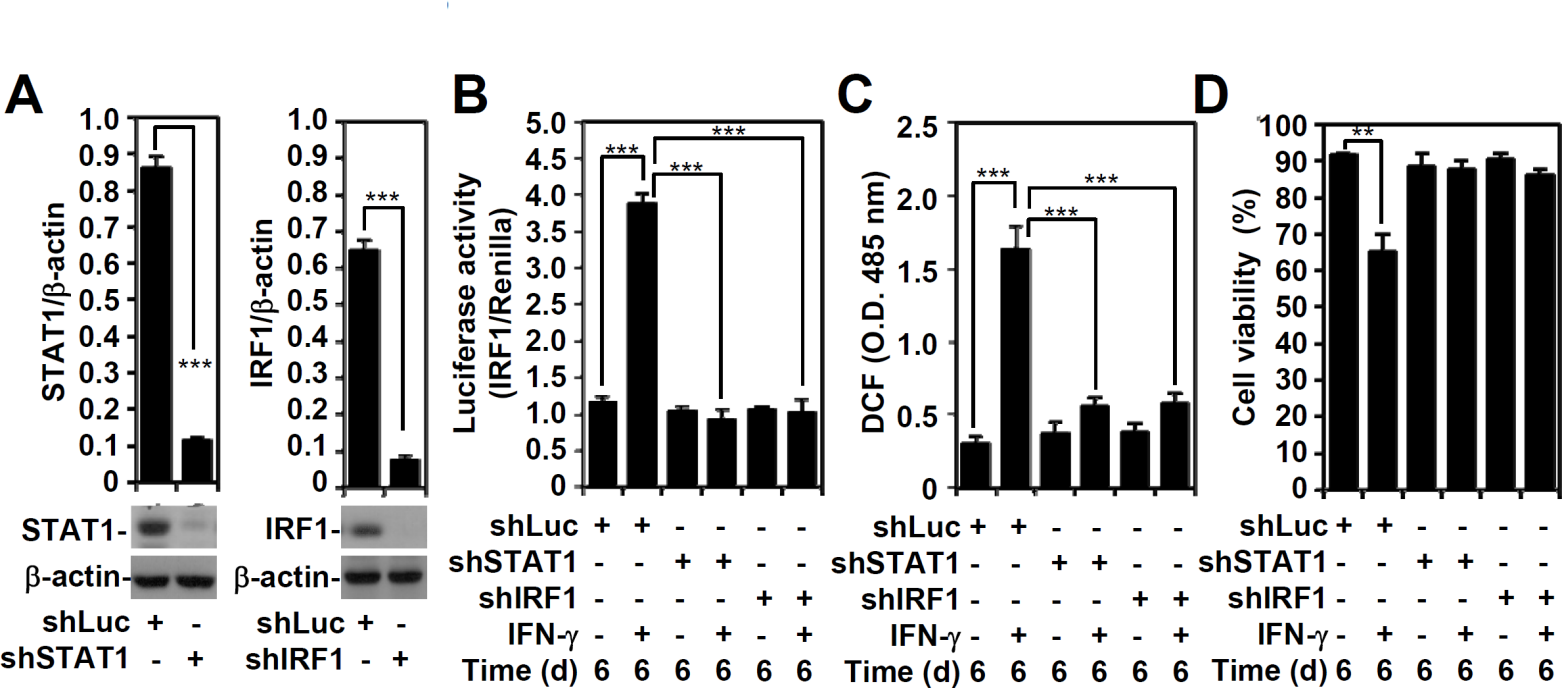
The effects of STAT1 and IRF1 on IFN-γ-induced transactivation of the *IRF-1* promoter and on IFN-γ-induced ROS generation. (A) A representative Western blot of the indicated proteins from A549 cells transfected with shRNA targeting luciferase (*shLuc*) and shRNA targeting STAT1 (*shSTAT1*) and IRF1 (*shIRF1*). β-actin was used as an internal control. The relative ratios of STAT1, IRF1, and β-actin are shown as the mean ± SD of three independent experiments. ***P < 0.001. (B) A luciferase reporter assay showed transactivation of *IRF-1* in IFN-γ (10 ng/ml)-treated shLuc-, shSTAT1-, or shIRF-1-transfected A549 cells for 6 days. The ratio of IRF-1 to the Renilla control is shown, and the data are presented as the mean ± SD from three independent experiments. ***P < 0.001. (C) ROS generation in A549 cells was determined using CM-H_2_DCFDA staining followed by analysis using a fluorescent plate reader. The data are the mean ± SD of triplicate cultures and are shown as relative optical densities (O.D.). ***P < 0.001. (D) The trypan blue exclusion test was performed to assess cell viability. The data are presented as the mean ± SD of triplicate cultures and are shown as relative percentages. **P < 0.01.

Using a lentiviral-based shRNA approach to investigate the effect of IFN-γ signaling regulation on ROS generation, we examined the involvement of IFN-γ-activated STAT1 and IRF-1, which are reportedly involved in ROS generation [1] (Fig 3A). An IRF-1 reporter assay confirmed that STAT1 plays an essential role in *IRF-1* transactivation and that silencing STAT1 and IRF-1 significantly (*P* < 0.001) abolishes *IRF-1* transactivation (Fig 3B). In both STAT1- and IRF-1-silenced A549 cells, IFN-γ-induced ROS generation was significantly (*P* < 0.001) lower compared to the control cells (Fig 3C). Because IFN-γ-activated STAT1 and IRF-1 are required for IFN-γ-induced mimic ETosis in A549 cells [11], we confirmed that silencing STAT1 and IRF1 diminishes the IFN-γ-induced blockade of cell viability (Fig 3D). These results indicate that IFN-γ induces ROS generation, which is involved in the induction of mimic ETosis, through a mechanism involving a STAT1-IRF-1-regulated signaling pathway.

### ROS generation is required for IFN-γ-induced mimic ETosis

**Fig 4:**
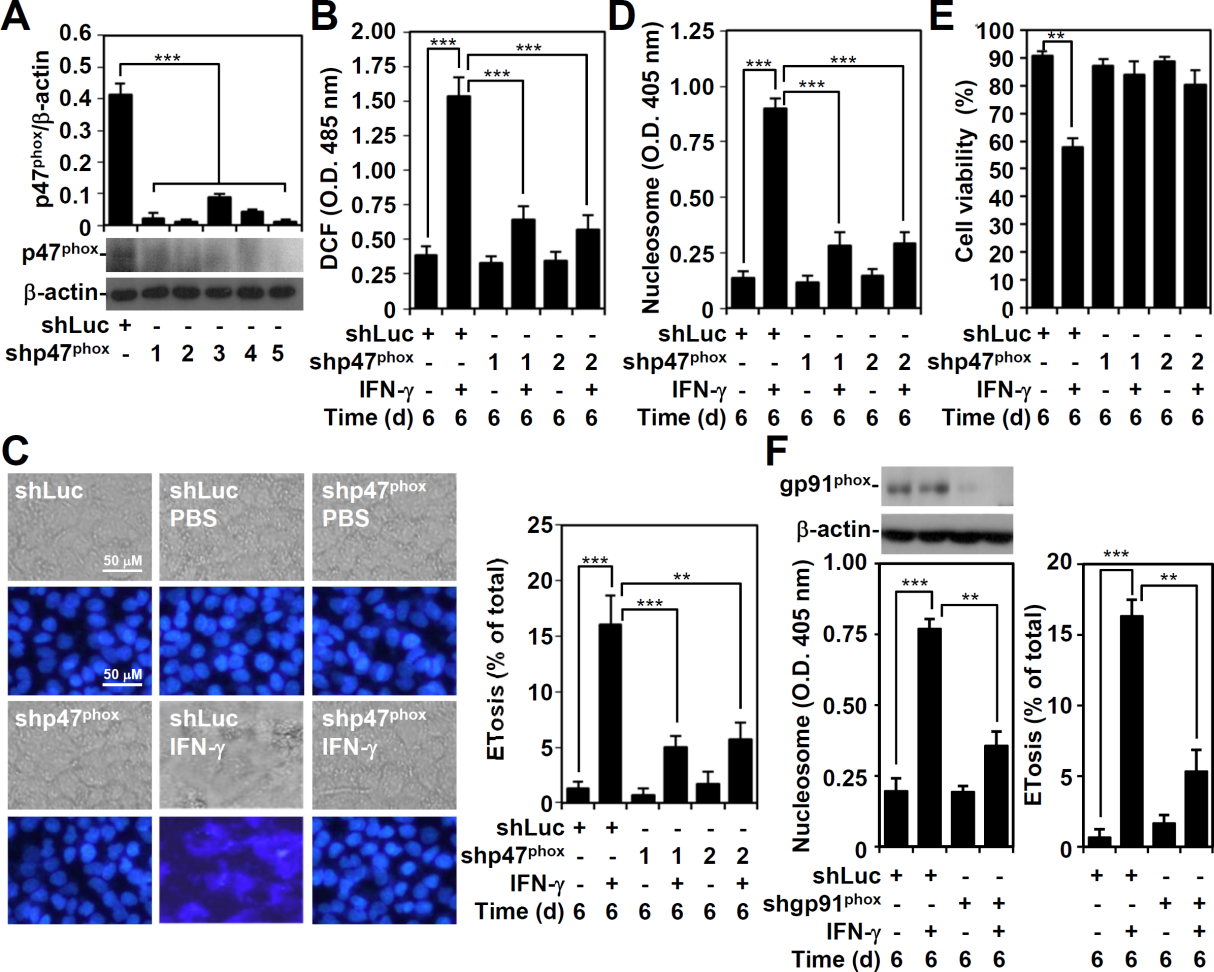
p47^phox^ mediates IFN-γ-induced ROS generation and a mimic ETosis. (A) A representative Western blot of the indicated proteins from A549 cells transfected with shRNA targeting luciferase (*shLuc*) and shRNA targeting p47^phox^ (*shp47*^*phox*^) (clones 1 to 5). β-actin was used as an internal control. The relative ratios of p47^phox^ and β-actin are also shown as the mean ± SD of three independent experiments. ***P < 0.001. (B) ROS generation in IFN-γ (10 ng/ml)-treated shLuc- and shp47^phox^ (clones 1 and 2)-transfected A549 cells for 6 days was determined using CM-H_2_DCFDA staining followed by analysis using a fluorescent plate reader. The data are presented as the mean ± SD of triplicate cultures and are shown as relative optical densities (O.D.). ***P < 0.001. Fluorescence microscopy was used to obtain representative images of DAPI-stained nuclei and to determine the percentage of cells with mimic ETosis (C). Nucleosome detection was measured using ELISA (D). The data for the percentages of cells with mimic ETosis and the O.D. value of nucleosome detection are shown as the mean ± SD of three independent experiments. **P < 0.01 and ***P < 0.001. (E) Cell viability was assessed using the trypan blue exclusion test. The data are presented as the mean ± SD of triplicate cultures and are shown as relative percentages. **P < 0.01. (F) shRNA targeting gp91^phox^ was used to silence gp91^phox^ as detected by Western blot analysis. The expression of nucleosome and the percentages of cells with mimic ETosis in gp91^phox^-transfected A549 cells with or without IFN-γ (10 ng/ml) treatment were determined accordingly. The data for the O.D. value of nucleosome detection and the percentages of cells with mimic ETosis are shown as the mean ± SD of three independent experiments. **P < 0.01 and ***P < 0.001.

p47^phox^ plays an important role in NADPH oxidase and ROS generation [22]. To investigate the role of NADPH oxidase-mediated ROS generation in IFN-γ-induced mimic ETosis, a lentiviral-based shRNA approach was used to silence p47^phox^ (Fig 4A). The shp47^phox^ clones 1 and 2 demonstrated a significant silencing ability (*P* < 0.001) by Western blotting and were used in this study. Notably, all shp47^phox^-transfected A549 cells showed significant (*P* < 0.001) resistance to IFN-γ-induced ROS generation (Fig 4B) and mimic ETosis, measured by DAPI staining (Fig 4C) and nucleosome detection (Fig 4D). Consistent with our previous study [11], cell viability assays confirmed the essential role of p47^phox^ in IFN-γ-induced cytotoxicity (Fig 4E). To confirm the role of NADPH oxidase, gp91^phox^ was also silenced by using shRNA and shgp91^phox^-transfected A549 cells not only showed a significant decrease in gp91^phox^ but also in IFN-γ-induced mimic ETosis as demonstrated by detecting nucleosome release and cells with mimic ETosis (Fig 4F). These findings show that ROS-generating NADPH oxidase is essential for IFN-γ-induced mimic ETosis.

### Regulation of IFN-γ-induced ROS generation for mimic ETosis induction

**Fig 5:**
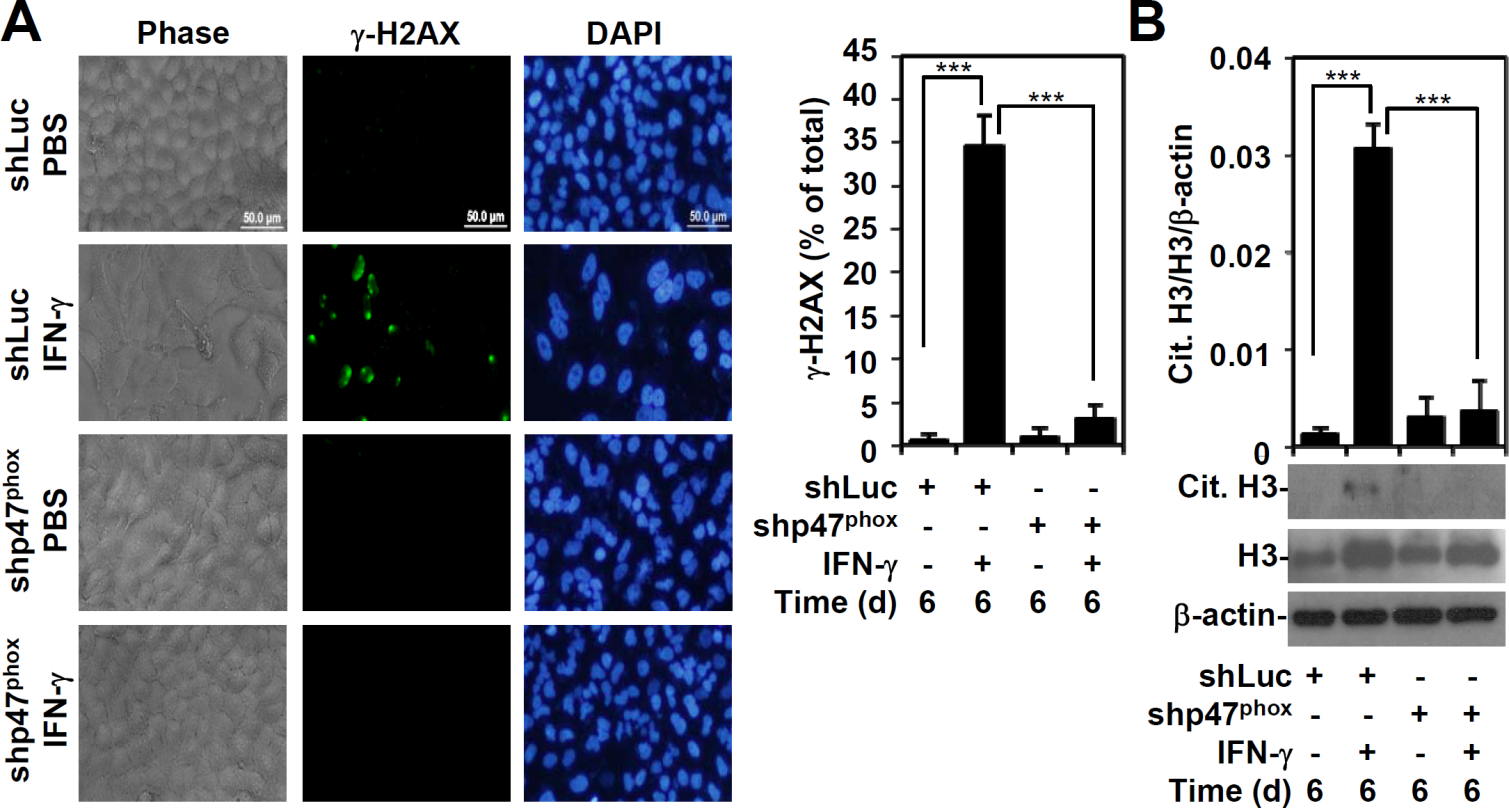
p47^phox^ is key for IFN-γ-induced DNA damage and histone H3 citrullination. (A) After IFN-γ (10 ng/ml) treatment in shLuc- or shp47^phox^-transfected A549 cells for 6 days, immunostaining with fluorescence microscopy was performed to determine the percentages of phosphor-γ-H2AX (Ser139)-positive nuclei. The data are shown as the mean ± SD of three independent experiments. ***P < 0.001. (B) After IFN-γ (10 ng/ml) treatment in shLuc- and shp47^phox^-transfected A549 cells for 6 days, Western blotting was performed to detect citrullinated histone H3 (Cit. H3) and histone H3. β-actin was used as an internal control. The relative ratios of Cit. H3 and β-actin are also shown as the mean ± SD of three independent experiments. ***P < 0.001.

We previously showed that IFN-γ induces DNA damage via caspase-3-mediated lamin A/C degradation and triggers DNA damage-associated ataxia telangiectasia and Rad3-related protein (ATR)/ataxia-telangiectasia mutated (ATM)-regulated mimic ETosis [11]. Because oxidative stress is involved in DNA damage [23], we hypothesize that IFN-γ-induced ROS generation is important for triggering the DNA damage that precedes mimic ETosis induction. As demonstrated through immunostaining, IFN-γ-induced phospho-γ-H2AX positivity was significantly (*P* < 0.001) lower in A549 cells with p47^phox^ silencing (Fig 5A). These studies highlight an important role for ROS in the IFN-γ-induced DNA damage response. One study showed PAD4-regulated histone H3 hypercitrullination and ETosis in neutrophils [24], which was also evident in A549 cells following DNA damage-associated ATR/ATM activation [11]. Furthermore, we showed that silencing p47^phox^ significantly (*P* < 0.001) reduced IFN-γ-induced histone H3 hypercitrullination (Fig 5B). These results demonstrate that IFN-γ induces DNA damage via a ROS-mediated pathway and triggers histone H3 hypercitrullination for mimic ETosis.

### IFN-γ-induced autophagy is independent of ROS generation

**Fig 6:**
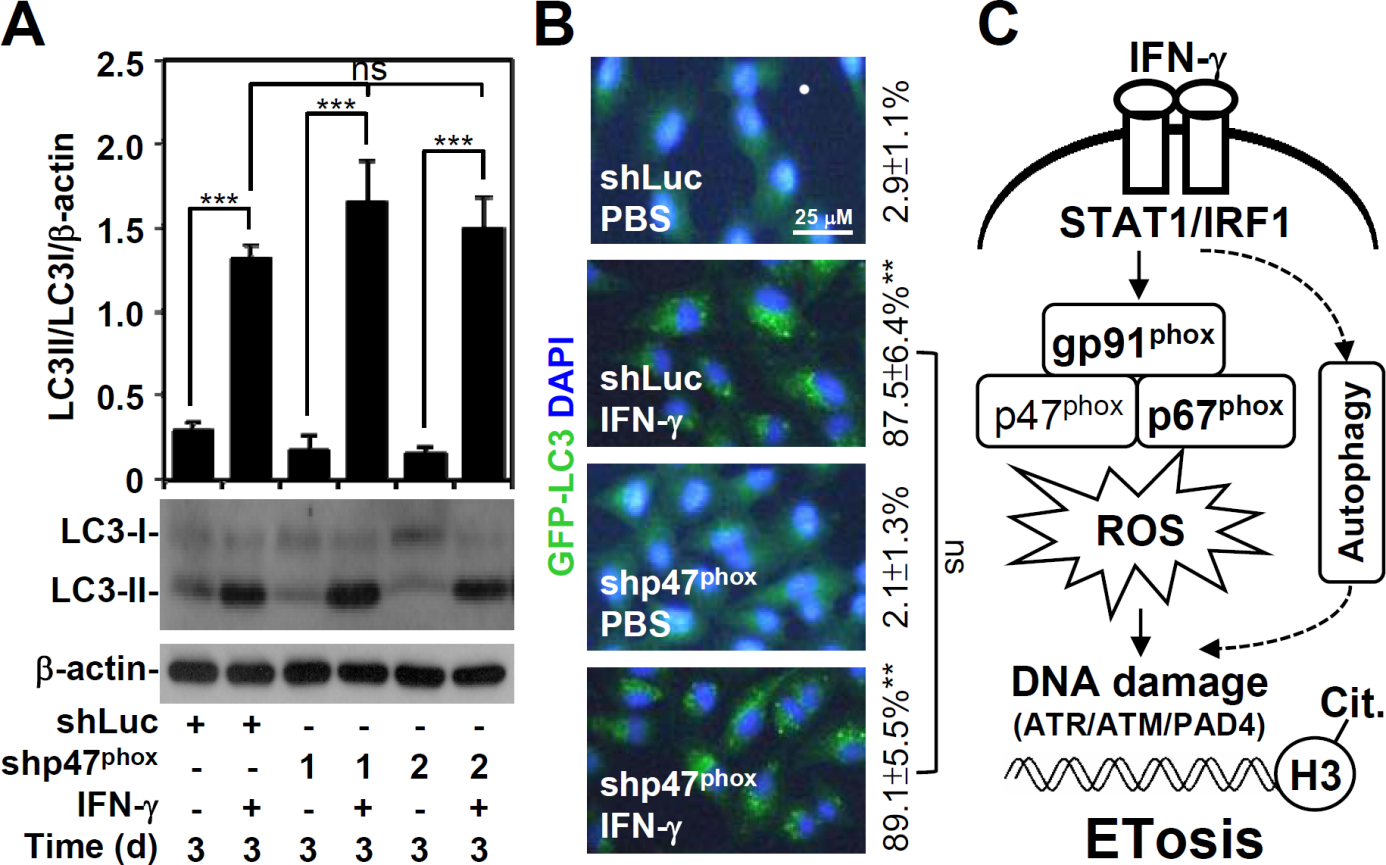
IFN-γ-induced autophagy is independent of oxidative stress. (A) After IFN-γ (10 ng/ml) treatment of shLuc- and shp47^phox^-transfected A549 cells for 6 days, Western blotting was performed to detect LC3-I/II expression. β-actin was used as an internal control. The relative ratios of LC3-II, LC3-I, and β-actin are also shown as the mean ± SD of three independent experiments. ***P < 0.001. ns, not significant. (B) Representative images of GFP-LC3 puncta formation captured by fluorescence microscopy. The data are shown as the mean ± SD obtained from three consecutive microscopic fields. **P < 0.01 compared to PBS. ns, not significant. (C) Schematic model of ROS-mediated IFN-γ-induced mimic ETosis in lung epithelial malignancy. IFN-γ induces autophagy, which causes DNA damage followed by ATR/ATM/PAD4-mediated histone H3 citrullination and a mimic ETosis. Additionally, IFN-γ-induced NADPH oxidase/ROS signaling enables DNA damage and a mimic ETosis.

Our current study shows that autophagy is required for IFN-γ-induced mimic ETosis [11]. Because ROS mediates autophagy under starvation conditions [25] and because ROS generation is required for IFN-γ-induced mimic ETosis, we investigated the involvement of IFN-γ-induced ROS generation in autophagy. However, p47^phox^ knockdown did not interfere with the LC-3 II conversion **(**Fig 6A**)** or with GFP-LC3 puncta formation **(**Fig 6B**)**, which are hallmarks of IFN-γ-induced autophagy [26]. These data demonstrate that ROS is not involved in IFN-γ-induced autophagy. Furthermore, IFN-γ concurrently induces autophagy and ROS generation to facilitate mimic ETosis in A549 cells.

## Discussion

IFN-γ may directly induce lung cancer cell death; however, the molecular mechanism remains unknown. Together with our previous study [11], this study demonstrates that IFN-γ induces growth inhibition, cytotoxicity, and mimic ETosis in A549 lung cancer cells. Mechanistic studies show that after IFN-γ treatment, IRGM- and ATF6-regulated autophagy promotes caspase-mediated lamin A/C disruption followed by DNA damage-associated ATR/ATM activation and the PAD4-mediated citrullination of histone H3 to induce mimic ETosis [11]. In this study, we show that IFN-γ treatment concurrently induces a NADPH oxidase-related ROS generation to cause DNA damage followed by citrullination of histone H3 and mimic ETosis (Fig 6C). Previous studies have demonstrated that ROS mediates ETosis in neutrophils [16, 17]; however, the underlying mechanism remains unclear. Importantly, our study provides evidence of the effects of NADPH oxidase/ROS signaling on DNA damage, resulting in IFN-γ-induced mimic ETosis.

For innate immunity, IFN-γ induces ROS production to kill intracellular bacteria in phagocytes [27] through the expression of NADPH oxidase components, such as gp91^phox^ and p67^phox^ [20, 21]. Recent studies have shown that IFN-γ induces ROS production in non-immune cells, and ROS production contributes to IFN-γ-induced cell apoptosis [28–30]. IFN-γ signaling controls tumor development and cancer immunoediting by upregulating various genes, including anticancer and immunomodulation genes [31]. Based on our study, IFN-γ-triggered ROS production in A549 cells is also mediated by NADPH oxidase components. However, the mechanism of IFN-γ-induced NADPH oxidase activation remains unknown, though IFN-γ does induce an increase in gp91^phox^ and p67^phox^ expression. Additionally, a previous study indicated that cooperation between the transcription factors PU.1 and IRF-1 along with the recruitment of the CREB-binding protein by the IFN consensus-binding protein are necessary for gp91^phox^ and p67^phox^ upregulation [32]. Furthermore, STAT1 and IRF-1 cooperatively trigger the IFN-γ-induced transcription of the gp91^phox^ gene [33]. We hope to confirm whether these transcription factors induce NADPH oxidase component expression and ROS production. STAT1-activated IRF-1 is partly involved, as demonstrated in this study.

ETosis in neutrophils requires the induction of autophagy and ROS generation [16, 17]. Through transcriptional and post-transcriptional regulation of autophagy, ROS generation controls autophagy in response to oxidative stress [34]. Therefore, we hypothesized that ROS-mediated autophagy mediated IFN-γ-induced mimic ETosis. However, we found that ROS was required for IFN-γ-induced mimic ETosis through DNA damage but not for autophagy induction. In IFN-γ-induced mimic ETosis, we suggest that autophagy and ROS are concomitantly involved. Oxidative stress causes DNA damage by triggering checkpoint activation in the cell cycle [23]. We previously showed that ATR/ATM mediates mimic ETosis after DNA damage by regulating PAD4-mediated citrullination of histone H3 [11]. Basically, caspases are not required for ETosis in neutrophils caused by LPS and fMLP [35]. However, ETosis-related autophagy and ROS may inhibit caspase activation to switch cell apoptosis to necrosis during ETosis. Our investigation demonstrates that IFN-γ induces caspase-3-mediated lamin A/C degradation followed by DNA damage-associated ATR/ATM activation. ROS may induce DNA damage directly or indirectly by activating other proteins. Furthermore, lamin A/C proteins are cleaved in a caspase-3-dependent manner [11, 36]; ROS-regulated caspase-3 activation in IFN-γ-treated A549 cells is also possible. It remains unclear whether ROS can modulate PAD4 activation directly or indirectly.

ETosis is primarily found in immune cells, including neutrophils, eosinophils, monocytes, macrophages, and mast cells. Through STAT1-IRF-1-regulated NADPH oxidase expression followed by ROS generation, we found that ROS-mediated DNA damage may also contribute to mimic ETosis in IFN-γ-treated A549 cells. The signaling axis for ROS-DNA damage in PAD4-mediated histone H3 citrullination in immune cells should be investigated further. Together with our current study [11], this study confirms that mimic ETosis occurs in non-immune A549 lung cancer cells but not in nontransformed lung epithelial cells (data not shown). An important question for future studies is whether IFN-γ-induced mimic ETosis in lung cancer would be helpful for anticancer treatments or whether it would slow tumorigenesis.
